# Suppression of MR1 by human cytomegalovirus inhibits MAIT cell activation

**DOI:** 10.3389/fimmu.2023.1107497

**Published:** 2023-02-10

**Authors:** Caroline L. Ashley, Brian P. McSharry, Hamish E. G. McWilliam, Richard J. Stanton, Ceri A. Fielding, Rommel A. Mathias, David P. Fairlie, James McCluskey, Jose A. Villadangos, Jamie Rossjohn, Allison Abendroth, Barry Slobedman

**Affiliations:** ^1^ Infection, Immunity and Inflammation, School of Medical Sciences, Faculty of Medicine and Health, and the Charles Perkins Centre, The University of Sydney, Sydney, NSW, Australia; ^2^ School of Dentistry and Medical Sciences, Faculty of Science and Health, Charles Sturt University, Wagga Wagga, NSW, Australia; ^3^ Department of Microbiology and Immunology, The Peter Doherty Institute of Infection and Immunity, The University of Melbourne, Melbourne, VIC, Australia; ^4^ Department of Biochemistry and Pharmacology, Institute of Molecular Science and Biotechnology (Bio21), The University of Melbourne, Melbourne, VIC, Australia; ^5^ Division of Infection & Immunity, School of Medicine, Cardiff University, Cardiff, United Kingdom; ^6^ Infection and Immunity Program, Department of Microbiology, Monash Biomedicine Discovery Institute, Monash University, Melbourne, VIC, Australia; ^7^ Infection and Immunity Program, Department of Biochemistry and Molecular Biology, Biomedicine Discovery Institute, Monash University, Melbourne, VIC, Australia; ^8^ ARC Centre of Excellence for Innovations in Peptide and Protein Science, Institute for Molecular Bioscience, University of Queensland, Brisbane, QLD, Australia

**Keywords:** human cytomegalovirus, herpesvirus, MHC class I related protein-1, MR1, mucosal-associated invariant T cells, MAIT cells, immune modulation

## Abstract

**Introduction:**

The antigen presentation molecule MHC class I related protein-1 (MR1) is best characterized by its ability to present bacterially derived metabolites of vitamin B2 biosynthesis to mucosal-associated invariant T-cells (MAIT cells).

**Methods:**

Through in vitro human cytomegalovirus (HCMV) infection in the presence of MR1 ligand we investigate the modulation of MR1 expression. Using coimmunoprecipitation, mass spectrometry, expression by recombinant adenovirus and HCMV deletion mutants we investigate HCMV gpUS9 and its family members as potential regulators of MR1 expression. The functional consequences of MR1 modulation by HCMV infection are explored in coculture activation assays with either Jurkat cells engineered to express the MAIT cell TCR or primary MAIT cells. MR1 dependence in these activation assays is established by addition of MR1 neutralizing antibody and CRISPR/Cas-9 mediated MR1 knockout.

**Results:**

Here we demonstrate that HCMV infection efficiently suppresses MR1 surface expression and reduces total MR1 protein levels. Expression of the viral glycoprotein gpUS9 in isolation could reduce both cell surface and total MR1 levels, with analysis of a specific US9 HCMV deletion mutant suggesting that the virus can target MR1 using multiple mechanisms. Functional assays with primary MAIT cells demonstrated the ability of HCMV infection to inhibit bacterially driven, MR1-dependent activation using both neutralizing antibodies and engineered MR1 knockout cells.

**Discussion:**

This study identifies a strategy encoded by HCMV to disrupt the MR1:MAIT cell axis. This immune axis is less well characterized in the context of viral infection. HCMV encodes hundreds of proteins, some of which regulate the expression of antigen presentation molecules. However the ability of this virus to regulate the MR1:MAIT TCR axis has not been studied in detail.

## Introduction

Human cytomegalovirus (HCMV) is a betaherpesvirus that causes lifelong infection with latency and is endemic in both developed and developing countries (1, 2). Infected individuals often harbor multiple strains of HCMV, either as a result of concurrent infection or superinfection (3, 4) and seroprevalence generally increases with age (5, 6). HCMV carriage results in a dramatic reconfiguration of the immune system which, combined with its prevalence, makes it one of the leading non-heritable causes of immune variation (7, 8). Despite this, public awareness of HCMV and the risks associated with infection is low (9). In immunocompetent individuals HCMV infection is generally asymptomatic in healthy individuals but it can cause serious complications in the immunonaïve (neonates) (10, 11) and immunocompromised people (for example, immunosuppressed transplant recipients (12–14)).

Mucosal-associated invariant T (MAIT) cells are a subset of predominantly CD8^+^ T cells involved in the innate response to bacterial and viral pathogens. MAIT cells were initially labeled invariant due to their consistent expression of the T cell receptor (TCR) alpha chain Vα7.2-Jα33 (15–18), however it is now accepted that MAIT cell TCRs can contain Vα7.2 paired with either Jα33, Jα12 or Jα20 and they are therefore considered to be semi-invariant (17, 19). MAIT cells are involved in the surveillance of mucosal surfaces including the oral musoca (20), the female genital tract (21) and breast tissue (22). MAIT cells can be activated by ligands presented on MR1 (23) or by combinations of pro-inflammatory cytokines such as IL-12 and IL-18 (24). MAIT cells were first characterized in MR1-dependent control of bacterial infections (16, 21, 25–34) and more recently have been implicated in MR1-independent antiviral responses (24, 35–38).

MR1 is a non-classical MHC I-like molecule whose function is known to include presentation of vitamin B-derived metabolites to activate MAIT cells (23, 39). During HCMV infection, other non-classical MHC I-like molecules have been reported to be regulated by this virus. For example, in HCMV infected cells, human leukocyte antigen-E (HLA-E), an inhibitor of NK cell activation, binds and is stabilized by the HCMV gpUL40 leader peptide (40, 41). Conversely HLA-G, a non-classical MHC I-like molecule that functions as an NK activation inhibitor, is targeted for proteasomal degradation by HCMV gpUS10 in infected HeLa cells (42) and trophoblasts (43). MHC I chain-related proteins A and B (MICA and MICB) which are highly polymorphic stress-induced NKG2D ligands that are similar to the MHC I heavy chain but do not associate with β2M, are both targeted for downregulation by HCMV (44–48). Our previous demonstration of MR1 downregulation by herpesviruses, with a focus on HSV-1 and VZV (49, 50), also indicated that both HCMV and murine cytomegalovirus were capable of inhibiting MR1 surface expression, however the functional outcome and the role of specific gene functions in such regulation was not studied.

Here we report modulation of surface and total MR1 protein by HCMV infection, and implicate the HCMV US9 gene product as contributing to this phenotype. We also demonstrate the ability of HCMV infected cells to inhibit bacteria-induced activation of primary MAIT cells in a MR1-dependent manner.

## Methods

### Cell culture, viruses and *E.coli*

Primary human foreskin fibroblasts (HFs) (ATCC), 293-TREX cells (invitrogen), HEK293Ts (ATCC), human telomerase immortalised (hTERT) HFs (51, 52) (and CRISPR/Cas-9 derivatives) as well as HF and ARPE-19 cells overexpressing MR1 and MR1-GFP (49) were grown in DMEM media supplemented with 10% foetal calf serum (FCS) and penicillin streptomycin (100 units/mL). TCR α/β deficient Jurkat-76 engineered to express the MAIT TCR: Jurkat.MAIT cells (18, 39, 53) were propagated in RPMI 1640 media supplemented with 10% FCS and penicillin streptomycin (100 units/mL). Human peripheral blood mononuclear cells (PBMCs) were isolated by density gradient centrifugation with Ficoll-Paque PLUS (GE Healthcare) from donor buffy coats obtained through the Australian Red Cross Blood Service. For use in MAIT activation experiments PBMCs were cultured in supplemented RPMI 1640 (10% FCS, 100 units/mL penicillin streptomycin). Before PBMCs were cocultured with HFs they were washed in PBS and transferred to folate free RPMI 1640 media supplemented with 10% human serum and 100 units/mL penicillin streptomycin.

The bacterial artificial chromosomes (BAC) derived viruses based on the low passage HCMV strain Merlin, RCMV1111 (Merlin) and GFP expressing RCMV1158 (Merlin-GFP), were described previously (54). AD169-UK strain was described previously (55). Stocks of HCMV were generated by harvesting virus from the supernatant of infected HFs. Supernatant was collected when all infected HFs displayed cytopathic effect (CPE) then spun at 845 xg for 10 min to pellet cell debris, and a second centrifugation for 2h at 21875 xg to pellet the virus. Virus pellets were resuspended in fresh supplemented DMEM and stored at -80°C.

Ultraviolet (UV) irradiation of virus was performed by applying 720mJ/cm^2^ of UV light using a CL-1000 Ultraviolet Crosslinker (Analytik Jena). Successful inactivation of virus was determined by a lack of development of CPE after incubation of UV-irradiated virus with fresh monolayers of HFs. For infection experiments, viable or UV-irradiated virus was applied to cultures for 90 minutes before being washed off; this point immediately after removal of the virus was taken as time zero.

DH5α *E.coli* for use in Jurkat.MAIT and PBMC MAIT cell activation assays were grown by shaking at 37° overnight in LB broth then fixed in 1% formaldehyde for 3 mins, vortexing for the first 60s and last 30s (28, 56). Fresh *E.coli* cultures were grown and fixed for each assay.

### Generation of viral recombinants

The US9 HCMV deletion mutant was genrated by recombineering of Merlin BAC as descibed previously (54) using the following primers;

US9 SacB For: GCCGGCGTGAGCCAGCGTTACCCAACAGCAGCCCAGGCCGACGAGGAGGCGCAGCCACCGCCTCATGGCGGCTTCGCCAGCCTGTGACGGAAGATCACTTCG

US9 SacB Rev:

GGTGGATACGTCCCTGGGTCCGAGGTCGGCACCGCGCCACCGGAAGGACTTCACGGGAAGAAGAGGCTAAAGACGATTGACTGAGGTTCTTATGGCTCTTG

Delete US9 open reading frame (ORF):

GCCCAGGCCGACGAGGAGGCGCAGCCACCGCCTCATGGCGGCTTCGCCAGAGCCGCCGGCACCGCGGCCGGCCGCAGGAAGCCGCCCGGCGCGTCGTCTG

Recombinant adenoviruses (RAds) expressing C-terminally V5-tagged gpUS7 and gpUS9 were generated as described previously using recombineering technology (57). The codon optimised US9 ORF was synthesised by Genscript and the US7 ORF was amplified from the Merlin genome using gene specific primers (US7 FOR: AAGACACCGGGACCGATCCAGCCTGGATCCttgacatacggtaataccatgcgg, US7 REV: GAGCGGGTTAGGGATTGGCTTACCAGCGCT gcccttgacaggataggtcaaa.RAds were generated from transfected BACs in 293-TREX cells. RAds were amplified and titered in 293-TREX cells as described previously (57).

### Jurkat.MAIT activation assays

HFs were infected with HCMV in supplemented DMEM at MOI 10. 72 hours post infection (hpi) HFs were washed in PBS and partially fixed *E*.*coli* (1000 CFU/HF) was added in folate free RPMI 1640 supplemented with FCS and penicillin streptomycin. 4 hours later *E.coli* was removed by washing with PBS and LEAF-purified anti-MR1 neutralizing antibody (clone 26.5, BioLegend) or appropriate isotype control was added for 1 hour (5 µg/mL) in folate free RPMI supplemented with FCS and penicillin streptomycin. Jurkat.MAIT cells were added at a Jurkat.MAIT : HF ratio of 2:1 in folate free RPMI supplemented with FCS and penicillin streptomycin. 20 hours later Jurkat.MAITs activation was assessed by flow cytometry.

### PBMC MAIT cell activation assays

HFs were infected with HCMV for 48 hours. Partially fixed *E*.*coli* (1000 CFU/HF) was added to the infected HFs overnight. HFs were washed extensively in PBS to remove *E.coli* and if applicable LEAF-purified anti-MR1 neutralising antibody (clone 26.5, BioLegend) or appropriate isotype control was added for 1 hour in folate free RPMI 1640 supplemented with human serum and penicillin streptomycin. PBMCs resuscitated the day before were added at a PBMC : HF ratio of 2:1 in folate free RMPI 1640 supplemented with human serum and penicillin streptomycin. After 5 hours of coculture PBMCs were stained for analysis by flow cytometry.

### CRISPR/Cas-9

Genome editing using CRISPR/Cas-9 was performed with the dual-vector lentivirus GeCKO system as described previously (58). Briefly, Cas-9 expressing lentivirus was harvested from the supernatant (filtered, 0.45 μm pore size) of HEK293T cells transfected with the packaging plasmid pCMV8.91, expression plasmid lentiCas-9-Blast and envelope plasmid pMD2G; 50% confluent hTERT-HFs, chosen for their longevity, were transduced with this lentivirus in the presence of 5 μg/mL polybrene. Successfully transduced hTERTs were selected with 5μg/mL blasticidin. Next these Cas-9 hTERT HFs were transduced with a lentivirus expressing guide RNA (gRNA) specific for the desired target gene, in this case MR1. To generate a gRNA specific for MR1 the following pair of DNA oligomers were annealed and ligated into the lentiguide-Puro expression plasmid following Esp3I (BsmBI) digestion: 5’- CACCGGGATGGGATCCGAAACGCCC -3’ and 5’- AAACGGGCGTTTCGGATCCCATCCC -3’. The resulting gRNA (in bold) was designed to target MR1 (59). This expression plasmid was transfected into HEK293T cells with the same packaging and envelope plasmids as the Cas-9 expressing lentivirus (pCMV8.91 and pMD2G, respectively). The filtered (0.45 μm pore size) supernatant of these cells and applied to 50% confluent Cas-9 hTERT HFs in the presence of 5 μg/mL polybrene. Cells successfully transduced with the gRNA lentivirus were then selected for with 1 μg/mL puromycin. In order to create clones, selected cells were seeded into 96 well plates at the approximate concentration of 0.5 cells/well, minimizing the chance of two cells ending up in the same well. Plates were monitored for growth over a 3-week period.

### Immunoblotting

Cell lysates from MR1 overexpressing cells were harvested in cell lysis buffer (50 mM NaCl, 50 mM TRIS pH8, 1% IGEPAL, 1% Triton X-100) as described previously (49). Lysates were resolved by precast polyacrylamide gels (Biorad) before immunoblotting onto PVDF membranes. Membranes were probed with the designated primary antibodies in 3% BSA in PBST, followed by incubation with an appropriate horseradish peroxidase (HRP)-conjugated secondary antibody (all Santa Cruz Biotechnology). The following primary antibodies were utilized: anti-MR1 and anti-HLA-A, B, C (Abcam), anti-GFP, anti-V5 and anti-GAPDH (all Santa Cruz Biotechnology).

### Flow cytometry

Cells collected for analysis by flow cytometry were washed with PBS and stained for viability with Zombie NIR fixable dye (BioLegend). Surface staining followed with cells resuspended in FACS buffer (PBS supplemented with 1% FCS and 10 mM EDTA) and relevant antibodies added for 30 min at 4°C. Cells were acquired on a BD LSR II cytometer (BD Biosciences, USA). Single stained UltraComp eBeads™ were washed in FACS buffer and fixed in the same way as surface stained cells, were used as compensation controls. FCS files were exported and analysed in FlowJo Software (BD Biosciences, USA).

The following antibodies were used to assess activation by flow cytometry: MR1 (clone 26.5, conjugated to PE, BioLegend), MHC I (clone G46-2.6, conjugated to APC, BD Biosciences), MICA (clone 159227, conjugated to APC, R&D Systems), CD3 (clone SK7, conjugated to BUV395, BD), CD161 (clone HP-3G10, conjugated to PE/Dazzle™ 594, BioLegend), TCR Vα7.2 (clone 3C10, conjugated to PE/Cyanine7, BioLegend), CD8 (clone SK1, conjugated to AlexaFluor^®^ 700), CD69 (clone FN50, conjugated to BV421). Matched isotype control antibodies were used where appropriate. Statistical analysis was performed in GraphPad Prism; when using the two-way ANOVA with multiple comparisions, Sidak’s correction was employed when the variance of both data sets needed to be taken into account e.g. neutralizing antibody and isotype control or knockout and parental cells. Tukey’s correction was used when comparisions were made within one set e.g. mock vs. mock + *E.coli* vs. HCMV vs. HCMV + *E. coli* within the neutralizing antibody treated cells only.

## Results

### HCMV infection downregulates both cell surface and total MR1 protein expression

To determine any impact on cell surface and total MR1 during HCMV infection, we utilized a BAC derived virus based on the low passage Merlin HCMV strain engineered to express GFP driven by an internal ribosome entry site (IRES) downstream of the HCMV IE2 (Merlin-GFP) (10). GFP expression from this virus allows for differentiation between infected and bystander cells. This concept was of particular interest when examining regulation of MR1 during HCMV infection because the closely related molecule MHC I is downregulated on HCMV antigen positive cells but upregulated on bystander cells that do not stain for HCMV antigens (60, 61).

**Figure 1:**
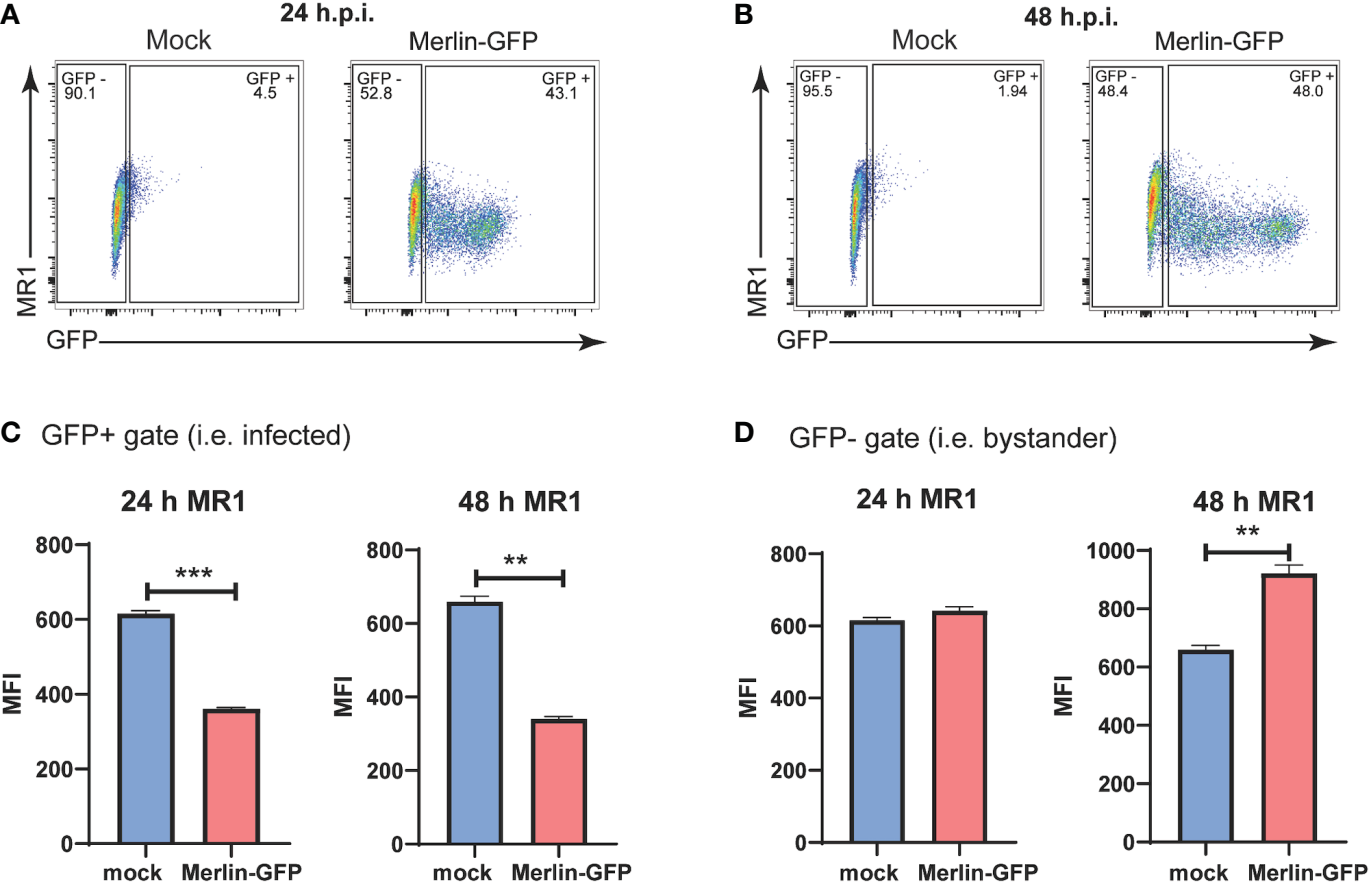
Downregulation of cell surface MR1 by HCMV. Primary HFS were infected with GFP expressing HCMV strain Merlin-GFP at an MOI of 3. 18 h prior to harvesting cells were treated with Ac-6-FP (2.5μM). At **(A)** 24 hpi and **(B)** 48 h.p.i. the expression of GFP and levels of cell surface MRI were assessed by flow cytometry. Using GFP expression as a measure of HCMV infection, the HFs were classified as GFP+ infected cells **(C)** or GFP- uninfected bystander cells **(D)**. Expression of cell surface MRI was analysed in each of these subsets. n = 3, error bars represent SEM, Students’ paired two-tailed t-test, p-value* <0.05, **<0.01, p ***<0.001.

Flow cytometry gates were established to separate GFP- bystander cells from GFP+ infected cells in cultures exposed to Merlin-GFP (**Figures 1A, B**). HFs were then assayed by flow cytometry for surface MR1 at 24 hpi and 48 hpi timepoints with cells treated with the MR1 ligand Ac-6-FP for the last 18h of the assay. Ac-6-FP binds MR1 in the ER, triggering a conformational change that facilitates association with β2m and trafficking to the cell surface (62). This analysis demonstrated that cell surface MR1 was significantly suppressed on the GFP+ infected cells at both 24 hpi and 48 hpi (**Figure 1C**). Levels of surface MR1 on the GFP- bystander cells were unchanged at 24 hpi but significantly elevated by 48 hpi (**Figure 1D**). The marked reduction of surface MR1 on HCMV infected cells suggests that, like MHC I, viral gene product(s) interfere with ligand-bound MR1 trafficking and/or stability, or targets it for degradation. The enhancement of MR1 surface expression on GFP- bystander cells at 48 hpi indicates that soluble factor(s) or defective virus particles that can bind to but not productively infect cells may mediate such an effect.

**Figure 2:**
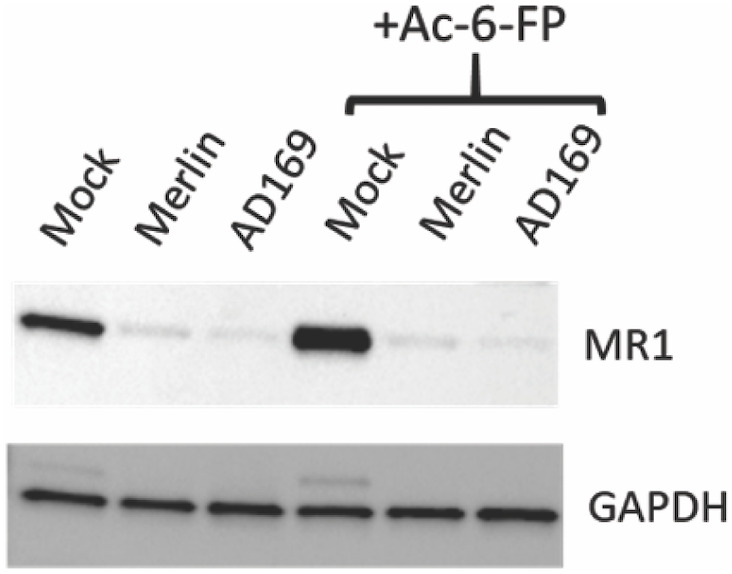
Downregulation of total cellular MR1 protein by multiple strains of HCMV. HFF-MR1 cells were mock infected, or infected with the HCMV Merlin or AD169 strains for 48 h before immunoblotting for MRI and GAPDH. The ligand Ac-6-FP (5 µM) was added at 16 h prior to harvesting for cell lysates for the indicated samples.

To determine if HCMV downregulated total cellular MR1 protein levels, HFs engineered to overexpress MR1 (49) were mock infected or infected with RCMV111 (Merlin) or lab-adapted HCMV strain AD169-UK. Cells were left untreated or treated with Ac-6-FP (5 μM) before immunoblotting for MR1 and GAPDH. Infection with either the Merlin or AD169 strains led to a potent reduction of total MR1 levels (**Figure 2**), consistent with the inhibition of cell surface levels as described here and previously (49). These data demonstrate a function conserved across HCMV strains downregulates MR1 expression levels and also show that the impact of HCMV infection on total MR1 expression is independent of MR1 ligand availability.

### The HCMV US9 gene product plays a role in suppressing cell surface MR1

To identify potential HCMV proteins regulating MR1 protein expression, interacting proteins were investigated using a co-immunoprecipitation and mass spectrometry-based approach (63). HF cells expressing MR1-GFP or control GFP were infected with HCMV for 48 h, before proteins were immuno-affinity isolated using the GFP tag, and interacting partners identified. A subset of HCMV proteins were specifically enriched within MR1-GFP isolations, with a top ranked HCMV candidate protein being gpUS9. This HCMV protein is a member of the US6 gene family. Members of this family are known to bind and regulate expression of other MHC and MHC-like molecules, with US9 itself recently identified to target surface expression of a specific allele of the NKG2D activating ligand MICA (MICA*008) (48, 64). For these reasons, we focused on the role of US9 in the context of potential modulation of MR1.

**Figure 3:**
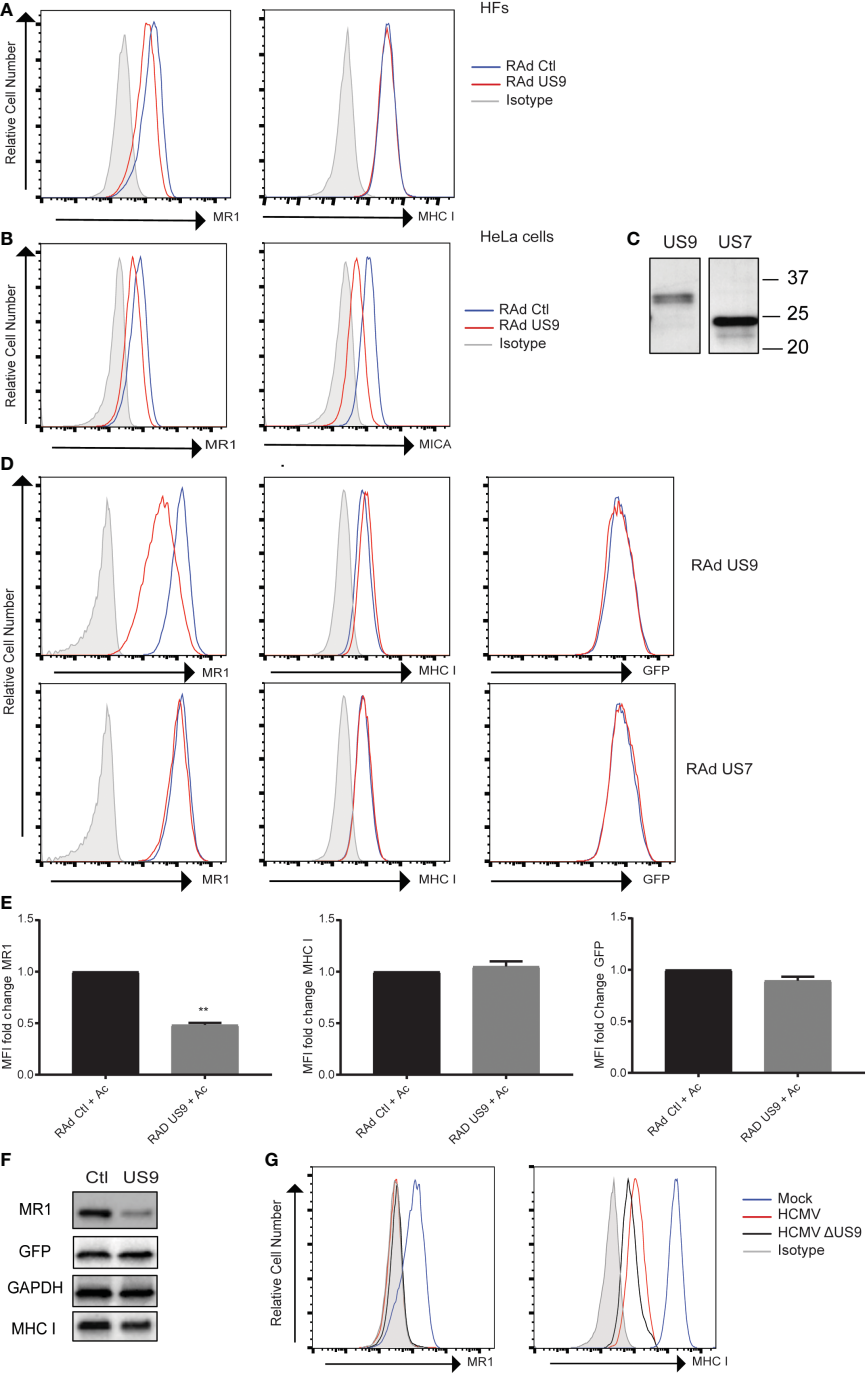
HCMV gpUS9 regulates MRI expression. **(A)** HFS or **(B)** HeLa cells were infected at an MOI of 100 with RAD US9 or RAd Ctl, with 5 Ac-6-FP (Ac) added 16 h prior to harvesting before staining for surface MRI, MHC I, MICA or isotype control at 48 h p.i. **(C)** Lysates harvested from Rad US9 or Rad US7 infected cells were immunoblotted with anti-V5 Ab **(D)** ARPE-19 MR1 cells were transduced with Rad US9 or Rad US7 as indicated, treated with 5 µM Ac-6-FP 16 h prior to harvesting, before staining for surface MRI or MHC I Rad Ctl (blue) or RADUS9/US7 (red) or isotype staining (grey) are indicated. **(E)** The fold change in MRI, MHC I and GFP MFI +/- SEM compared to RAD Ctl are graphed (n = 3). **p<0.01. **(F)** Lysates harvested from ARPE-19 MRI cells transduced with RAd Ctl or RAD US9 were immunoblotted for MR1, GFP, MHC I and GAPDH. **(G)** HFS were infected with HCMV or HCMV AUS9 at an MOI of 10, treated with 5 µM Ac-6-FP 16 h prior to harvesting before staining for surface MRI, MHC I at 48 h.p.i., infected cells gated on MHC low cells.

The ability of US9 to regulate MR1 surface expression was assayed using a replication deficient adenovirus (RAd) expressing US9. HFs transduced with RAd US9 or control Rad were treated with Ac-6-FP then stained for surface MR1 or MHC I (**Figure 3A**). There was a limited but consistent reduction in MR1 surface levels but not MHC I in cells expressing US9. This effect of gpUS9 on MR1 surface expression was recapitulated in HeLa cells which are homozygous for the MICA*008 allele where gpUS9 expression could also limit MICA surface expression as previously described (48), confirming functional expression of US9 (**Figure 3B**).

To determine if the capacity to target MR1 was a common feature of US6 family members, the ability of gpUS7 to regulate MR1 was also assayed. Initially we confirmed expression of gpUS9 and gpUS7 from the RAds by immunoblotting (**Figure 3C**) before testing their effect in MR1 overexpressing cells (ARPE-19 MR1). These cells express MR1 and GFP from the same expression cassette *via* an internal ribosomal entry site (IRES) upstream of the GFP open reading frame (49). MR1 surface expression was also significantly reduced in ARPE-19 MR1 cells by gpUS9, however the related protein gpUS7 was not capable of modulating MR1 surface expression (**Figures 3D, E**). Levels of MR1 expression were also reduced in gpUS9 expressing cells as measured by immunoblotting, mirroring the effect on cell surface expression, with MHC I and GFP levels unchanged with gpUS9 expression (**Figure 3F**). gpUS9 expression also significantly reduced MR1 surface levels in MR1-GFP fusion protein expressing cells with GFP fluorescence (a readout of total MR1 levels in this setting) also reduced (**Supplementary Figure 1**) consistent with MR1 immunoblotting detailed in **Figure 3F**.

To test the ability of gpUS9 to regulate MR1 in the context of HCMV infection we assayed a US9 deletion mutant, based on the Merlin strain. However loss of US9 expression failed to rescue MR1 expression (**Figure 3G**), suggesting that additional functions reside in the HCMV genome to potentially target MR1. This is unsurprising given that HCMV often encodes multiple gene functions to target the same pathway e.g. the downregulation of MHC I on infected cells can be attributed to the actions of multiple HCMV genes that either target it for degradation (e.g. US2, US11 (65–67)), interfere with peptide loading (e.g. US6 (68–70)) or disrupt trafficking from the ER to cell surface (e.g. US3 (66)) (reviewed in (71)). Indeed, although US9 is capable of reducing MICA*008 surface expression in isolation, a HCMV deletion mutant lacking US9 is still capable of reducing MICA*008 surface levels (48) consistent with the recent definition of pUL147A as an additional HCMV encoded immunevasin targeting MICA*008 (47).

### Productive HCMV infection inhibits expression of cell surface MR1 in the presence of *E.coli* derived MAIT cell activating MR1 ligand

While the synthetic ligand Ac-6-FP is useful to study the modulation of surface MR1 by HCMV infection because it stably upregulates MR1 (31, 72–74), it does not activate MAIT cells (39, 74). To assess the functional consequences of ligand bound MR1 regulation by HCMV infection the source of MR1 ligand was changed to partially fixed *E.coli* (a known driver of MAIT cell activation), described previously (75). Primary HFs were infected with Merlin or AD169 strains at an MOI of 10 and harvested for analysis of surface MR1 expression by flow cytometry at 24 hpi and 48 hpi with the partially fixed *E.coli* added for the last 18 hours of the assay. Cells were also treated with UV-irradiated HCMV. UV irradiation damages viral DNA, generating virus particles capable of binding and entry but unable to initiate *de novo* gene expression or replicate allowing determination of the requirement for *de novo* viral gene expression in the regulation of MR1.

**Figure 4:**
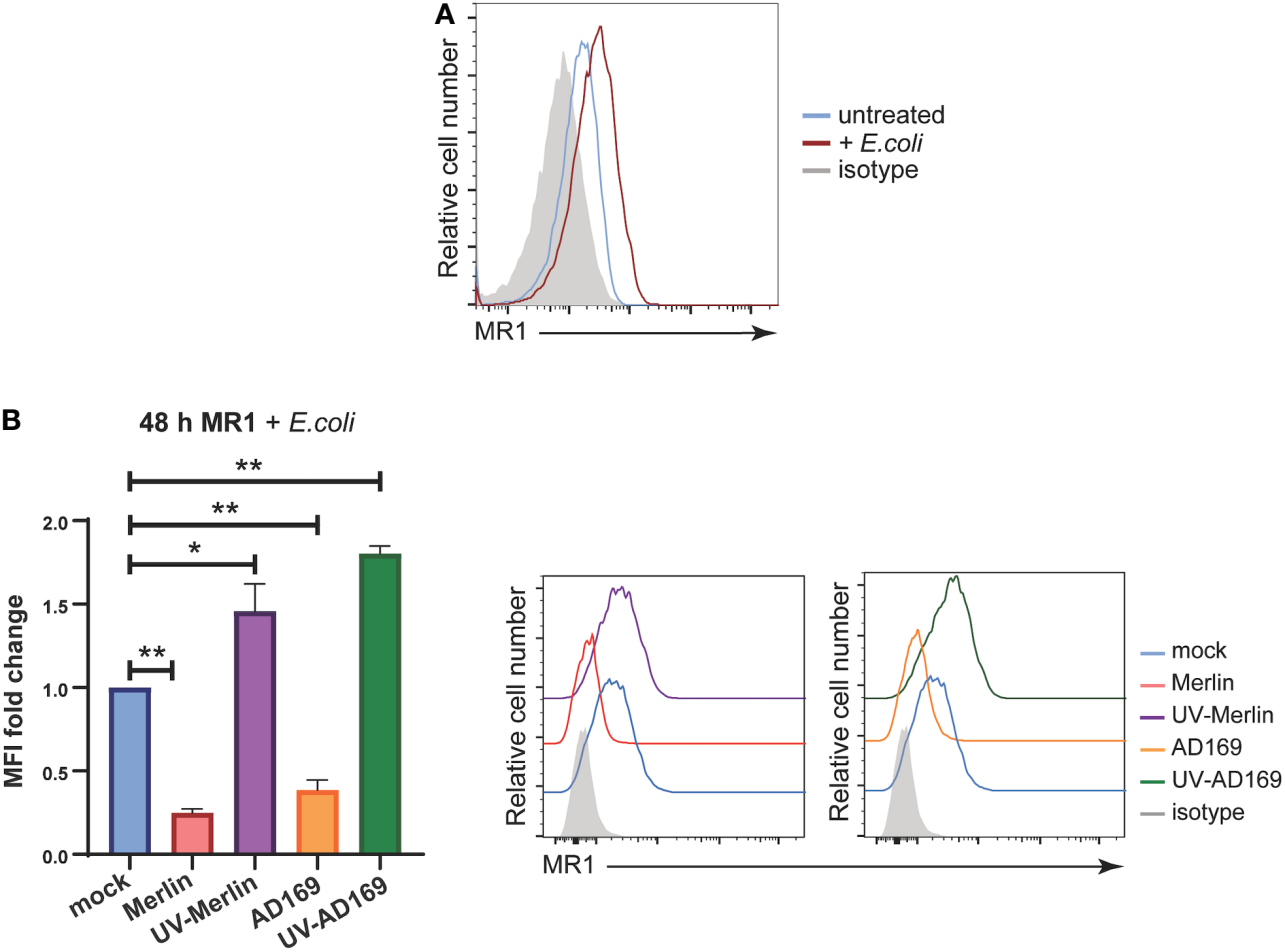
HCMV infection significantly inhibits *E. coli*-induced MR1 upregulation while UV-HCMV enhances it. **(A)** Primary HFs were treated with 1000 CFU/cell of partially fixed *E. coli* or left untreated for 18 h before being stained for analysis of cell surface MRI by flow cytometry. **(B)** Primary HFS were infected with HCMV (either Merlin or AD169) or treated with UV-HCMV (either Merlin or AD169) at an MOI of 10. 30 h.p.i cells were treated with 1000 CFU/cell of partially fixed *E*. *coli* then 18 later stained for analysis of cell surface MRI by flow cytometry. Infected cells are gated for as MHC I low. Statistical significance was determined by two-way ANOVA, n = 3 error bars represent SEM, p-value* <0.05, **< 0.01.

Treatment of HFs with *E. coli* for 18 hours resulted in upregulation of surface MR1 as expected (**Figure 4A**) (75). This expression of surface MR1 in response to *E.coli* was significantly inhibited in primary HFs infected with either Merlin or AD169 strains (**Figure 4B**). Interestingly, MR1 surface expression was enhanced on cells treated with UV-Merlin or UV-AD169 compared to mock treated cells (**Figure 4B**) consistent with the upregulation of MR1 seen in bystander cells (**Figure 1**). This demonstration of MR1 downregulation during productive HCMV in the presence of a biologically relevant source of MR1 ligand suggests that HCMV-mediated MR1 downregulation may have functional consequences for microbe driven MAIT cell activation.

### HCMV infection inhibits MAIT TCR activation

To determine whether a functional consequence of HCMV mediated suppression of MR1 was impaired activation of the MAIT TCR, HFs were mock infected or infected with HCMV Merlin or AD169 for 48 hours. These cells were then exposed to partially fixed *E.coli* for 4 hours, and then cocultured with Jurkat.MAIT cells. These cells are immortalized Jurkat T cells, which naturally lack the αβ TCR, that have been transduced to express a MAIT TCR (18, 39, 53). Jurkat.MAIT cells have been used previously to assess MR1-dependent activation (18, 39, 49, 53). The activation profile of Jurkat.MAIT cells was then determined by flow cytometry using the activation marker CD69.

**Figure 5:**
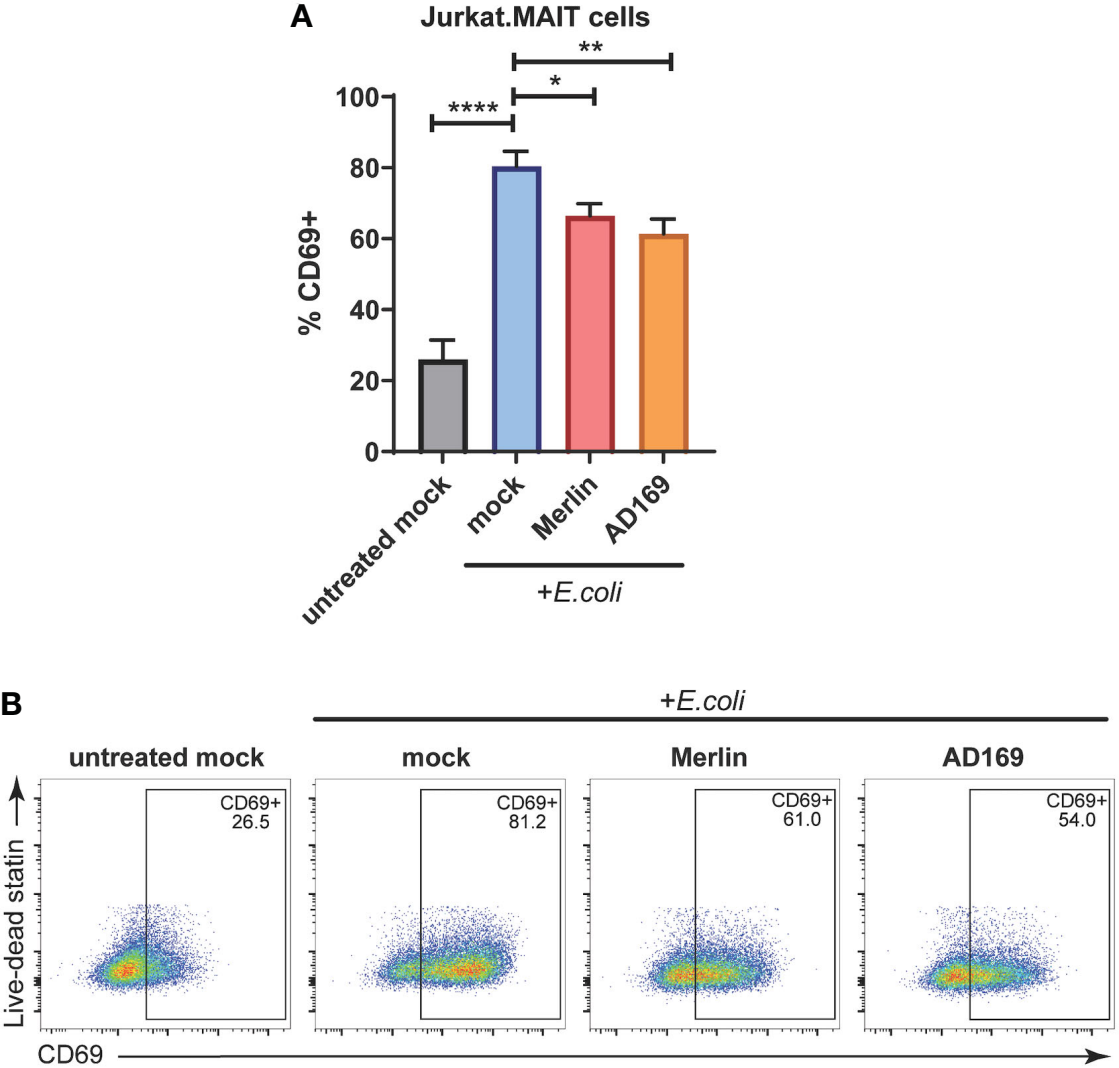
HCMV inhibits E.coli-induced Jurkat.MAIT cell activation. **(A)** Primary HFs were mock infected or infected with HCMV strain MERI111 or HCMV strain AD169 at an MOI of 10. 48 h.p.i. cells were treated with 1000 CFU/cell of partially fixed *E.coli*. After 4 h the ligand source was washed off. After 1 h Jurkat.MAIT cells were added to the HFS in folate-free RPMI medium at a ratio of 2:1. Cells were cocultured overnight before the Jurkat.MAIT cells were harvested for analysis by flow cytometry with their activation assessed by surface CD69 expression. Error bars represent SEM, n = 3, *p<0.05, two-way ANOVA, Dunnett's multiple comparisons to mock + *E.coli*. **(B)** Representative dot plots showing % Jurkat.MAIT CD69+. **<0.01, p ****<0.0001.

As determined by CD69 cell surface expression, exposure of Jurkat.MAIT cells to *E. coli* treated HFs resulted in significant activation (**Figures 5A, B**). However, when the HFs were infected with HCMV Merlin or AD169 strains prior to treatment with *E.coli*, they were significantly less able to activate Jurkat.MAIT cells than their mock infected, *E. coli* treated counterparts (**Figures 5A, B**). These data indicate that *E. coli* driven activation of Jurkat.MAIT cells is significantly reduced by both Merlin and AD169 strains of HCMV.

### HCMV infection inhibits MR1-dependent activation of primary human blood-derived MAIT cells

**Figure 6:**
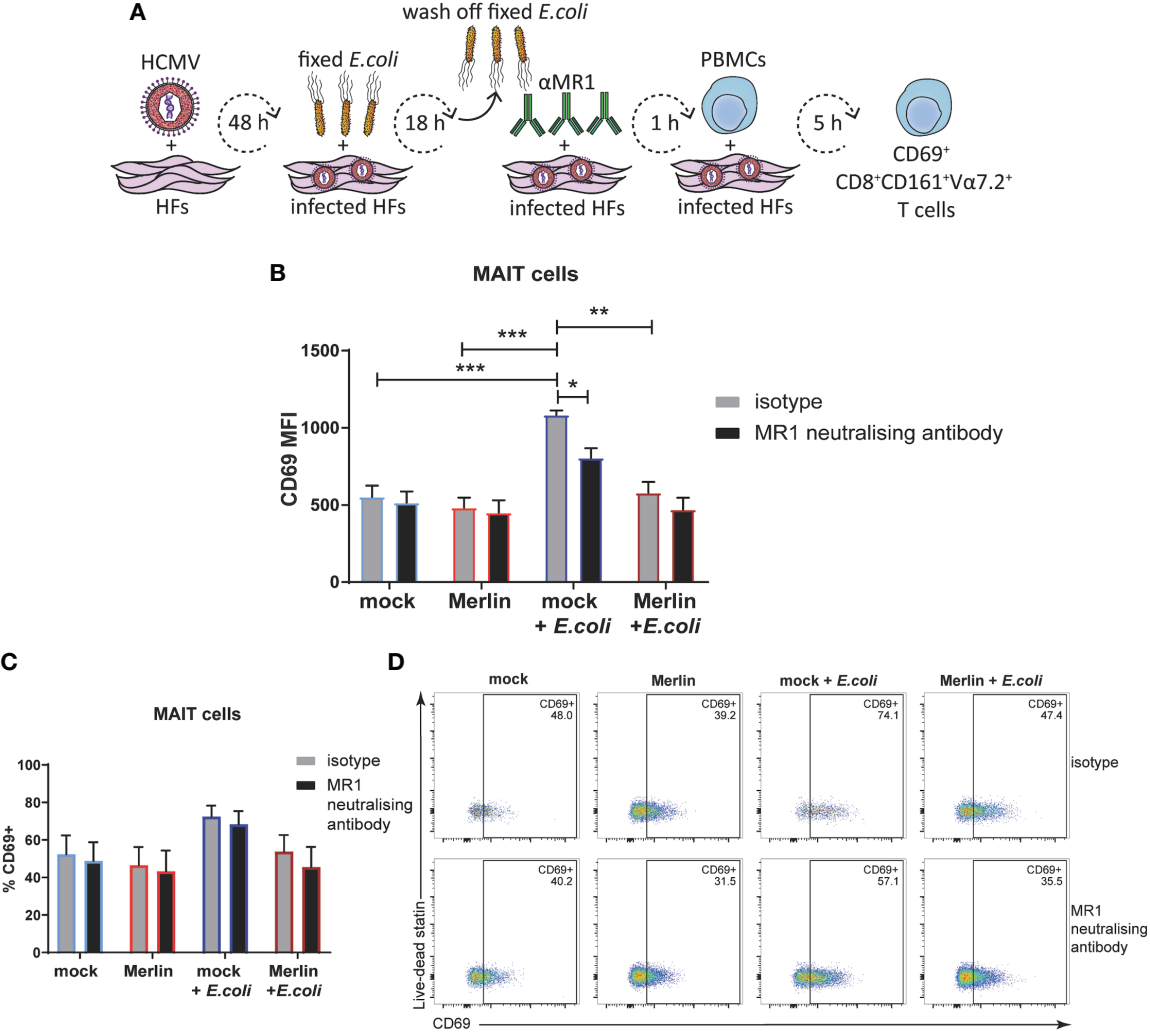
HCMV infection inhibits *E. coli*-induced MRI-dependent primary MAIT cell activation. **(A)** Primary HFS were infected with HCMV strain Merlin at an MOI of 20, and treated with partially fixed *E. coli* before the addition of MRI neutralising antibody and coculture with whole PBMCs in folate free RPMI medium as depicted **(A)**. Mock infected HFS were subjected to the same treatment and mock or infected HFS that had not been treated with partially fixed *E. coli* were analysed in parallel. Levels of surface CD69 on CD3+CD8+CD161+Vα7.2 MAIT cells were assessed by flow cytometry using MFI **(B)** and % CD69+ **(C)**, representative dot plots shown in **(D)**. Two-way ANOVA, n 3, error bars represent SEM. Sidak's multiple comparisons for MRI neutralising antibody vs. isotype, Tukey's multiple comparisons for mock vs HCMV vs mock + *E. coli* vs. HCMV + *E. coli* * p<0.05, ** p<0.01, ***p<0.001.

To extend analyses of HCMV-mediated MR1 downregulation to primary cell activation, the ability of HCMV infection to impair activation of primary human PBMC-derived MAIT cells was assessed. HFs were mock infected or infected with HCMV (Merlin) for 48 hours before being exposed to partially fixed *E. coli* for 18 hours and then cocultured with PBMC for 5 hours. MAIT cells were identified by flow cytometry as being positive for cell-surface CD3, CD8, CD161 and the invariant TCR chain Vα7.2 (15, 76, 77), and their activation state was determined by co-staining for CD69. **Figure 6A** depicts this experimental approach. As MAIT cells can also be activated by multiple viral infections in an MR1-independent manner (predominantly by IL12 and IL18) (37, 78, 79), MR1 neutralising antibody or its isotype control were included in this assay to determine the degree of MR1-dependence.

When using median fluorescence intensity (MFI) to measure the degree of MAIT cell activation, there was no significant difference in the MAIT activating capacity of mock infected or HCMV infected primary HFs in the absence of *E. coli* (**Figure 6B**). However, *E.coli* treated, mock infected cells significantly activated primary MAIT cells and this activation was inhibited by MR1 neutralizing antibody (**Figure 6B**). HCMV infection also inhibited *E.coli* driven MAIT cell activation consistent with efficient targeting of MR1 surface expression by viral infection (**Figure 6B**). When % CD69+ was used to assess MAIT cell activation all significance was lost (**Figures 6C, D**), suggesting that in this assay set up both HCMV infection and the MR1 neutralizing antibody were affecting the degree of activation more significantly than the proportion of activated MAIT cells. Together, these results demonstrate impairment of bacterial-driven, MR1-dependent MAIT cell activation by HCMV infection.

### Inhibition of primary MAIT cell activation by HCMV infection is comparable to knockout of MR1 expression

To further examine the ability of HCMV to inhibit MR1-dependent MAIT cell activation, MR1 knockout fibroblasts were engineered using CRISPR/Cas-9 technology. The dual-vector lentivirus GeCKO system (58) was used to transduce human telomerase immortalized (hTERT) HFs first to express Cas-9 and then with a MR1 specific gRNA. A MR1 knockout Cas-9 hTERT HF clone was identified by screening for surface MR1 expression following treatment with MR1 ligand Ac-6-FP and loss of ability to activate Jurkat.MAIT cells following treatment with 5-OP-RU (**Supplementary Figure 2**).

**Figure 7:**
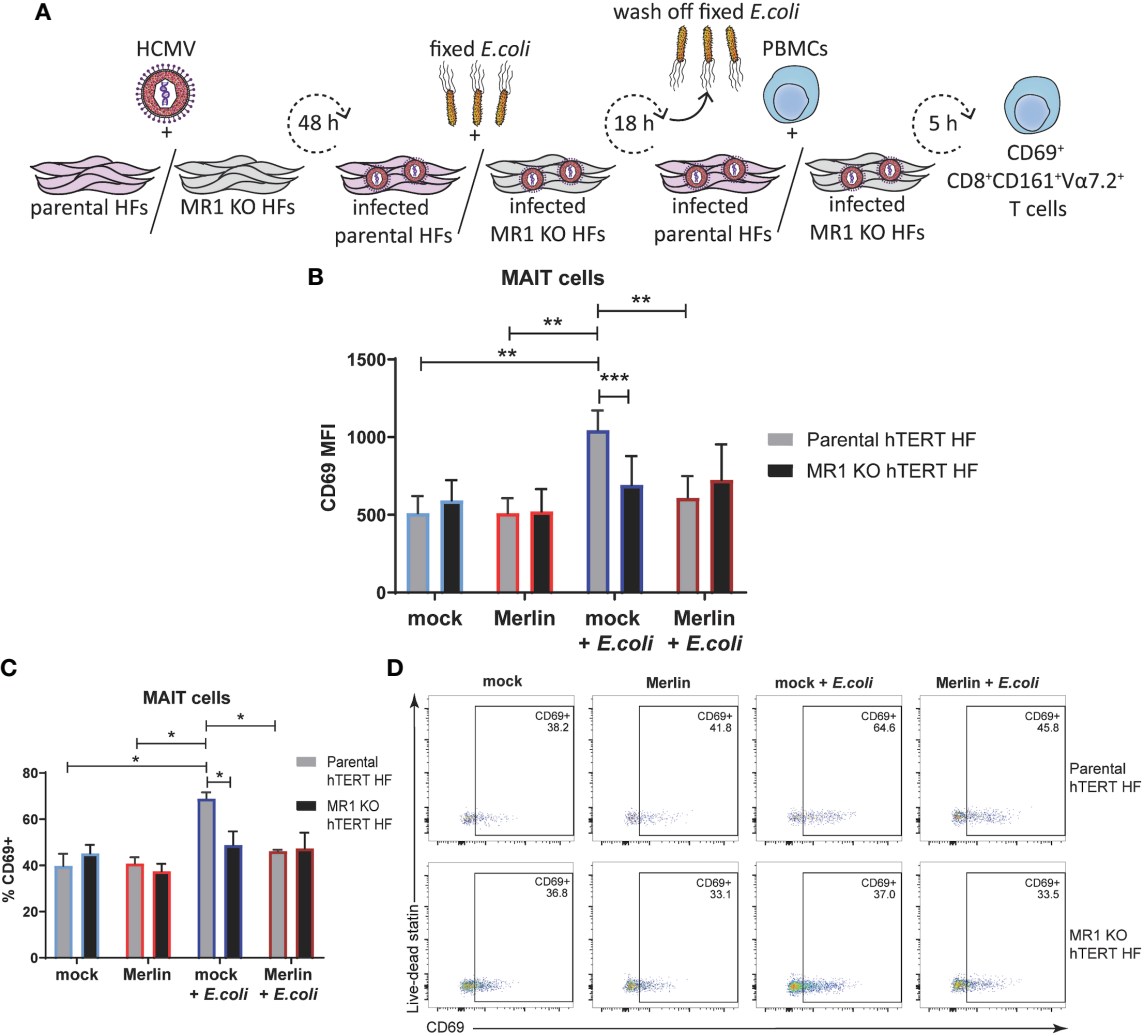
The inhibition of primary MAIT cell activation during HCMV infection is comparable to that achieved by MRI knockout using CRISPR/Cas-9. **(A)** Parental Cas-9 hTERT HFS and a successful MR1 knockout clone 2 were infected with HCMV strain MER1111 at a MOI of 20. At 48 h.p.i cells were treated with partially fixed *E. coli* overnight. Cells were washed to remove the *E. coli* and whole PBMCs were added for 5 h in folate free RPMI medium. In parallel, cells were mock infected and/or not treated with *E. coli*. Levels of surface CD69 on CD3+CD8+CD161+Vα7.2 MAIT cells were assessed by flow cytometry using MFI **(B)** and % CD69+ **(C)**, representative dot plots shown in **(D)**. n = 4. error bars represent SEM, two-way ANOVA, Sidak's multiple comparisons for MRI KO vs. parental hTERT HFS. Tukey's multiple comparisons for mock vs HCMV vs mock + *E. coli* vs. HCMV + *E. coli* ** p<0.01, ***p<0.001.

The primary MAIT cell activation assay was then performed in parental Cas-9 hTERT HFs in parallel with MR1 knockout cells (MR1 KO hTERT HFs) as illustrated in **Figure 7A**. Flow cytometry assessment of surface CD69 revealed no significant difference in the MAIT activating capacity of mock infected or HCMV infected parental or MR1 KO cells HFs in the absence of *E. coli* (**Figure 7B**) consistent with the requirement of MR1 ligand to drive activation. However, *E.coli* treated, MR1-expressing parental cells, activated primary MAIT cells and this activation was significantly reduced in MR1 KO cells (**Figure 7B**). HCMV infection of parental cells also inhibited *E.coli* driven MAIT cell activation in parental cells (**Figure 7B**) consistent with efficient targeting of MR1 surface expression by viral infection. When % CD69+ was used to examine at the proportion of activated MAIT cells in this assay, the significant relationships observed with CD69 MFI were preserved (**Figures 7C, D**), suggesting that the MR1 neutralizing antibody used in **Figure 6** was insufficient to prevent MR1-dependent MAIT cell activation for the entire 5 h coculture. Together, these results confirm impairment of bacterial-driven, MR1-dependent MAIT cell activation by HCMV infection. Thus, HCMV infection suppresses MR1-dependent activation of primary MAIT cells.

## Discussion

This study builds upon our previous reports identifying that suppression of MR1 by viral infection is a feature of herpesvirus infection (49, 50). Here we define the regulation of MR1 expression by multiple HCMV strains. HCMV infection was capable of efficiently suppressing MR1 surface expression driven by either synthetic MR1 ligand or provided by treatment with bacteria having an intact vitamin B2 biosynthesis pathway. We identified a specific HCMV-encoded glycoprotein, gpUS9, that was capable of targeting the surface expression of MR1 and decreasing total MR1 protein. However, the generation and use of a US9 deletion mutant suggested that there are multiple MR1 targeting functions encoded by the virus, as this mutant virus still efficiently suppressed MR1 surface expression. The functional outcome of this MR1 regulation was tested using Jurkat.MAIT cells as well as primary PBMC derived MAIT cells. Using both MR1 neutralizing antibody and cells engineered to knockout MR1 expression we confirmed that HCMV could efficiently inhibit MAIT TCR dependent activation in an MR1-dependent fashion.

gpUS9 is a glycoprotein that is a member of the US6 gene family of HCMV. A number of functional activities have been previously ascribed to gpUS9 including regulation of type I interferon responses by targeting MAVS and STING (80), regulation of cell to cell spread in epithelial cells (81, 82) and most relevant to this study, targeting of a specific allele of the MHC-like protein, MICA*008 (48, 64). Here we used a recombinant adenovirus vector to demonstrate that gpUS9 was capable of inhibiting MR1 but not MHC I surface expression. We also confirmed the targeting of MICA*008 by gpUS9. gpUS9 expression in isolation lead to a specific reduction in total MR1, using both untagged and GFP tagged MR1 mirroring the reduction in MR1 levels demonstrated following HCMV infection. A HCMV US9 deletion mutant based on the Merlin strain was generated however this virus was still capable of efficiently inhibiting MR1 expression. This is unsurprising given that HCMV encodes a number of functional proteins to target the same pathway e.g. MICA (44, 45, 47, 48), MHC I (71), FcγR activation (83). We predict that HCMV encodes additional gene product(s) to target MR1 that will be a focus of future studies.

gpUS9 targets MICA*008 for proteasomal degradation with the N-terminal signal peptide recently implicated as playing a key role in the specific targeting of MICA*008 (48, 64). Although we identified a reduction in total MR1 protein levels with gpUS9 expression the specific pathway leading to such regulation was not studied. Additional studies will focus on how gpUS9 regulates MR1 levels as well as identifying the importance of individual domains of the protein in controlling MR1 expression.

The best characterized role of MAIT cells to date in responding to viral infection is through virus induced, cytokine mediated activation of MAIT cells, independent of the MR1:TCR axis (24, 78, 79). Here we have described the specific targeting of MR1 by HCMV. Therefore, in the absence of any known virally expressed/induced MR1 ligand, we tested the capacity of HCMV to regulate bacteria-driven MR1-dependent MAIT cell activation. Using partially fixed *E. coli* as an activating stimulus, HCMV was capable of efficiently inhibiting MAIT cell activation using both primary MAIT cells and reporter Jurkat.MAIT cells. To confirm that such regulation was dependent on MR1, two approaches were tested, namely genetic ablation of MR1 by CRISPR/Cas-9 technology as well as MR1 neutralization by specific antibodies. Both approaches identified that HCMV suppression of MAIT TCR dependent activation was MR1 dependent.

In the context of allogeneic hematopoietic stem cell transplantation (HSCT), where HCMV reactivation from latency is a frequent and often life-threatening complication in HSCT recipients, increased HCMV replication has been linked to development of acute and chronic graft vs. host disease (GVHD) (84, 85), and poor MAIT cell reconstitution in HSCT transplant recipients has been linked to the development of acute and severe GVHD (86–88). These findings suggest that there could be an association between HCMV reactivation post-HSCT and poor MAIT cell reconstitution. Indeed, we have reported that MAIT cell levels at the initial detection of HCMV reactivation in HSCT recipients are significantly lower in patients who subsequently go on to develop high-level reactivation requiring antiviral intervention, compared to those who go on to develop low-level, self-limiting reactivation (89). Additionally, HCMV infection in HSCT patients can be correlated with increased incidence of bacterial and fungal infections (90, 91), which may indicate suppression of MAIT cell function. While there is still more to be done in the context of natural HCMV infection and MAIT cells, these observations raise the possibility that HCMV may impact on, or be impacted by, the MAIT cell response during clinically significant infection and/or co-infection.

## Data availability statement

The raw data supporting the conclusions of this article will be made available by the authors, without undue reservation.

## Ethics statement

The studies involving human participants were reviewed and approved by University of Sydney Human Research Ethics Committee. Written informed consent for participation was not required for this study in accordance with the national legislation and the institutional requirements.

## Author contributions

CA, BM, and RM performed experiments. CA, BM, HM, RM, AA, and BS designed experiments and/or analyzed data. CA, BM, AA, and BS wrote the manuscript. HM, RS, CF, DF, JM, JV, and JR provided reagents and/or key planning. All authors contributed to the article and approved the submitted version.
