# Supplementary figures and images for: Suppression of MR1 by human cytomegalovirus inhibits MAIT cell activation
Source: Front Immunol. 2023 Feb 10;14:1107497. doi: 10.3389/fimmu.2023.1107497 (PMC9950634; doi:10.3389/fimmu.2023.1107497)

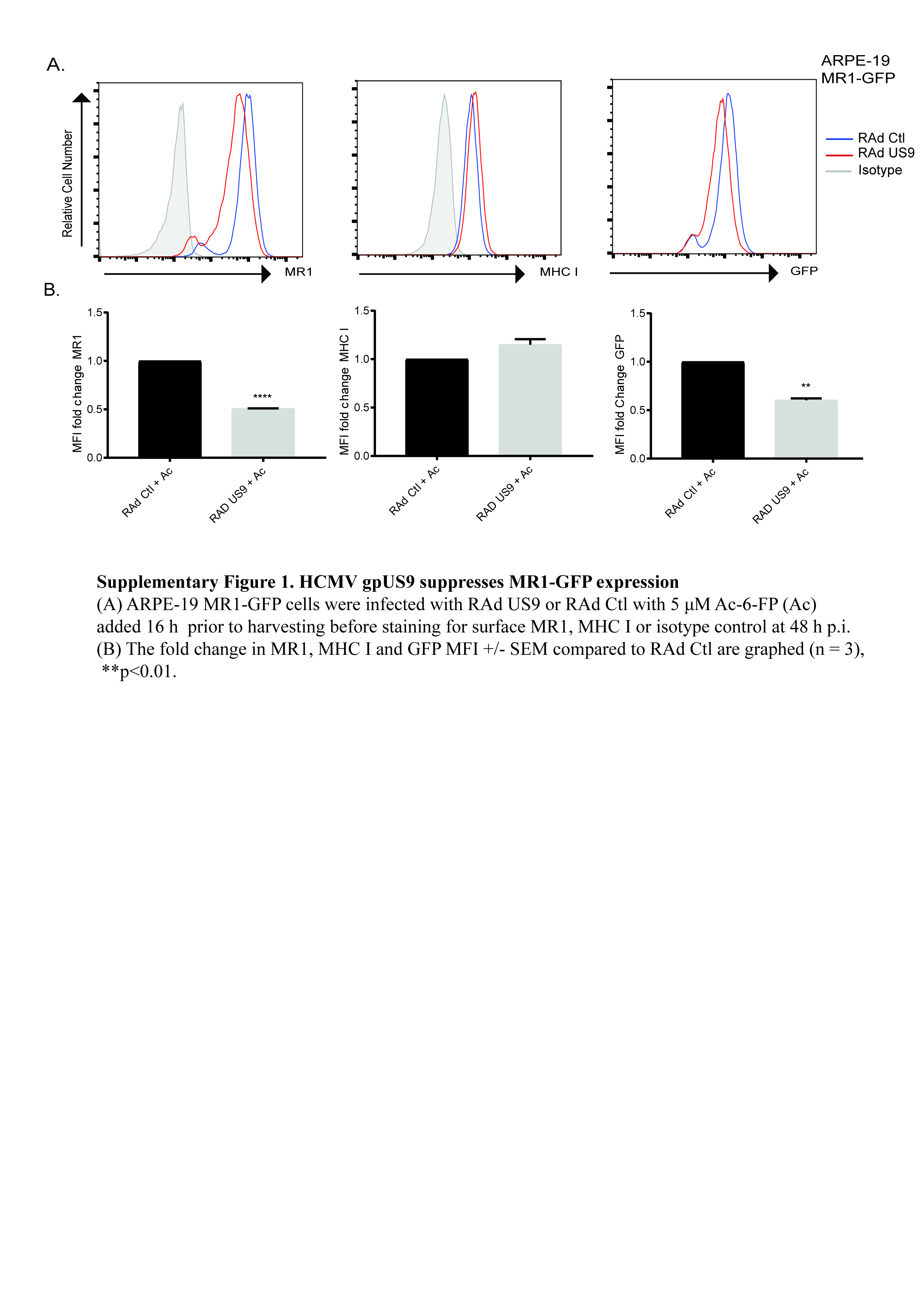

Supplement: Supplementary file 1 [file Image_1.tif]

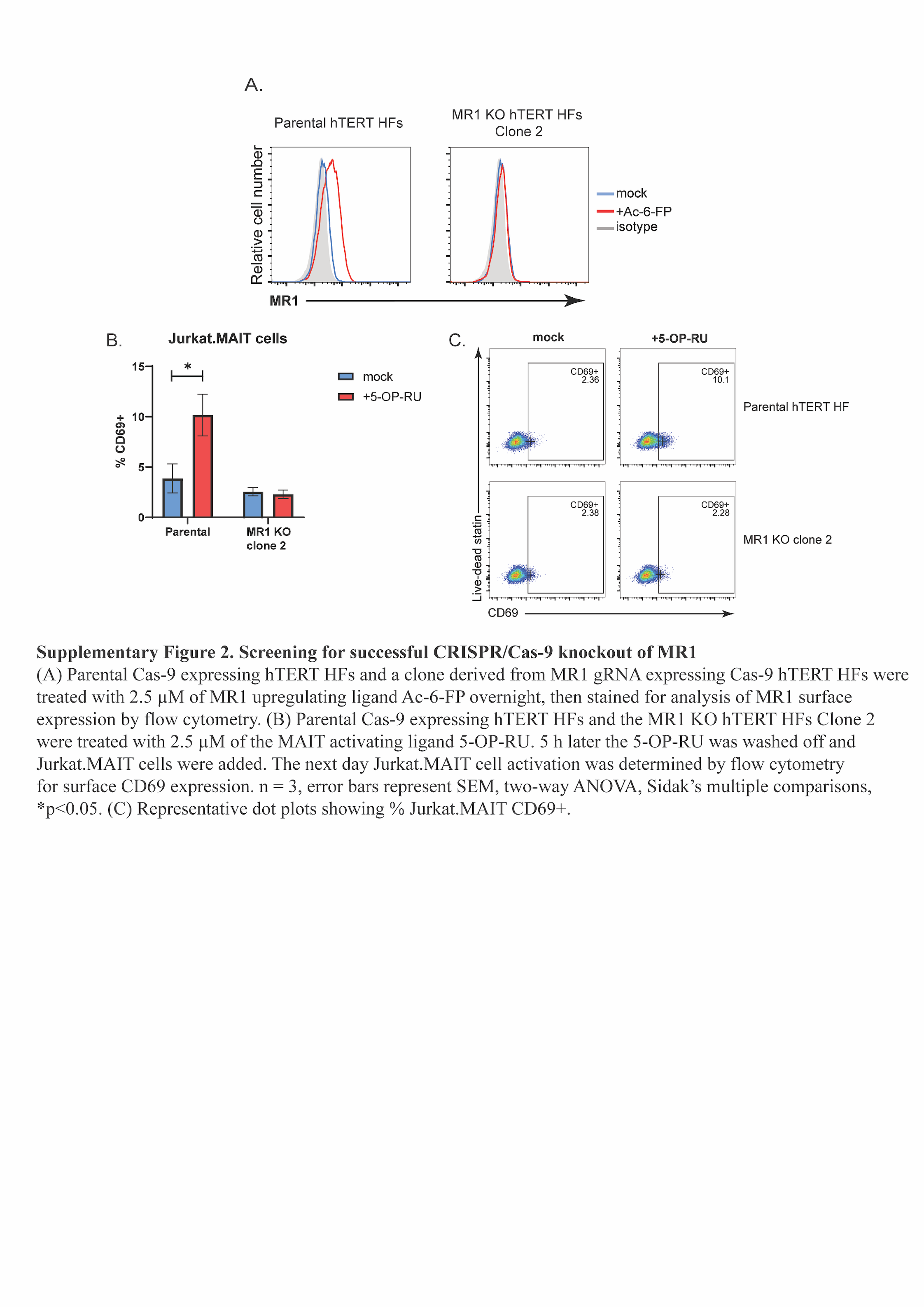

Supplement: Supplementary file 2 [file Image_2.tif]
